# Nemesis - a molecular modeling package

**DOI:** 10.1186/1758-2946-5-S1-P12

**Published:** 2013-03-22

**Authors:** Jakub Štěpán, Petr Kulhánek, Milan Lenčo, Zora Střelcová, Aleš Křenek, Jaroslav Koča

**Affiliations:** 1 CEITEC − Central European Institute of Technology, Masaryk University, 62500 Brno, Czech Republic; 2 National Centre for Biomolecular Research, Masaryk University, 611 37 Brno, Czech Republic; 3 Institute of Computer Science, Masaryk University, 602 00 Brno, Czech Republic

## 

Very important part of theoretical simulations is a process of structure preparation, geometry editing and detailed examination of properties. Here we present advanced molecular modeling package called Nemesis [1]. The software is mainly focused on the molecular structure preparation for calculations, taking into account typical user problems. Nemesis is open-source, multi-platform, modular, intuitive and easy to use. The package functionality is exhibited to end-users *via *projects. Currently, four project types are supported - *Sketch Structure, Build Structure, Collaboration*, and *Trajectory*.

The first mentioned, *Sketch Structure *project offers simple structure drawing in 2D. It is based on limited functionality of the Ketcher editor [2]. More advanced structure building is offered by the *Build Structure *project. This project is intended for building structures from predefined 3D fragments or single atoms, it offers also tools for typical user tasks as changing of atomic charge, bond order, names, connectivity, or translations, rotations and copying of atoms or atom groups. Prepared structures can be optimized by various force fields with imposition of geometrical restraints. Structure topologies and geometries can be easily altered and/or measured. The package fully supports an import and export of various molecular formats using OpenBabel library [3] and internal frameworks. Several graphical representations can be used to visualize structures and their properties. The *Collaboration *project extends the *Build Structure *project by tools necessary for collaborative structure modeling and visualization in group of users. Users can share the same view of modeled structure in real time and structure changes are propagated to all users in real time as well. Finally, the *Trajectory *project can be used to analyze results from molecular dynamics simulations or geometry optimization and coordinate driving performed by external programs.

The package is written in C++ programming language. Its functionality can be extended via plugins. We are intensively working on extensions focused on RMSD calculation, scripting possibilities, or advanced work with biomolecules containing standard residues. We also continuously add and extend structure visualization models and abilities.

## References

[B1] Nemesis https://lcc.ncbr.muni.cz/whitezone/development/nemesis

[B2] Ketcher, GGA software services, Cambridge, USA http://www.ggasoftware.com

[B3] O'Boyle NM Banck M James CA Morley C Vandermeersch T Hutchison GR J Cheminf 2011 3 33 10.1186/1758-2946-3-33 PMC3198950 21982300

